# Supplementary material for: Attention deficit in children with attention deficit hyperactivity disorder at primary school age measured with the attention network test (ANT): A protocol for a systematic review and meta-analysis
Source: PLoS One. 2022 Oct 25;17(10):e0275599. doi: 10.1371/journal.pone.0275599 (PMC9595530; doi:10.1371/journal.pone.0275599)
Supplement: S1 Checklist — (DOC) [file pone.0275599.s001.doc]

**PRISMA-P (Preferred Reporting Items for Systematic review and Meta-Analysis Protocols) 2015 checklist: recommended items to address in a systematic review protocol***

| Section and topic | Item No | Checklist item |
| --- | --- | --- |
| ADMINISTRATIVE INFORMATION | | |
| Title: |  |  |
| Identification | 1a | Attention deficit in children with attention deficit hyperactivity disorder at primary school age measured with the attention network test (ANT): a protocol for a systematic review and meta-analysis |
| Update | 1b | NaN |
| Registration | 2 | In accordance with the guidelines, our systematic review protocol was registered with the International Prospective  Register of Systematic Reviews(PROSPERO) on 14 May 2021 (registration number CRD42021249768). |
| Authors: |  |  |
| Contact | 3a | Author Affiliations1,2,* ;Małgorzata Lipowska 1,2; Łucja Bieleninik 1,3 ;Angel M. Dzhambov 4,5  1 Institute of Psychology, Faculty of Social Sciences, University of Gdansk, Gdańsk, Poland  2 Institute of Applied Psychology, Faculty of management and social communication, Jagiellonian University, Kraków, Poland  3 GAMUT-The Grieg Academy Music Therapy Research Centre, NORCE Norwegian Research Centre, Bergen, Norway  4 Department of Hygiene, Faculty of Public Health, Medical University of Plovdiv, Plovdiv, Bulgaria  5 Institute for Highway Engineering and Transport Planning, Graz University of Technology, Graz, Austria  *Correspondence: Gabriela Gradys: gabriela.gradys@gmail.com |
| Contributions | 3b | All authors were responsible and accountable for all parts of the work related to the protocol. More specifically, GG and ML had the original idea. GG and ŁB contributed to the conception and design of the protocol. GG performed the first literature searches. GG, ŁB and AD contributed to writing the manuscript. AD prepared the analysis plan. All authors revised the manuscript and approved the final version to be published. |
| Amendments | 4 | If we need to amend this protocol, we will give the date of each amendment, describe the change, and give the rationale in  this section. Changes will not be incorporated into the protocol. |
| Support: |  |  |
| Sources | 5a | This systematic review is funded by The "NeuroSmog: Determining the impact of air pollution on the developing brain” project, witch is carried out within the TEAM-NET program of the Foundation for Polish Science co-financed by the European Union under the European Regional Development Fund (Nr. POIR.04.04.00-1763) and statutory funds of the Institute of Psychology of University of Gdańsk. |
| Sponsor | 5b | NaN |
| Role of sponsor or funder | 5c | The funding body does not influence the design of the study and the writing of the manuscript. |
| INTRODUCTION | | |
| Rationale | 6 | For those diagnosed with ADHD, attention deficits are among the symptoms that have the most negative impacts on their everyday lives. The neurological basis for the occurrence of ADHD has not yet been fully described. The diagnostic criteria proposed by the American Psychological Association are written in such a way that suggests that ADHD may result from deficits of various attention attributes. No neuropsychological methods exist in specialist diagnostics that allow reliable distinctions to be made between primary attention disorders in ADHD and secondary problems that may arise due to other disorders, such as specific learning disorder or behavioural disorders like conduct disorder (CD) and oppositional defiant disorder (ODD). Primary attention deficits are caused by abnormal brain development, like in ADHD. Secondary attention deficits can appear during the lifespan because of unhealthy habits, such as suboptimal nutrition, long screen time or poor sleeping; other neurodevelopmental and psychiatric disorders, challenging life experiences, and brain injury. The distinction between primary and secondary attention deficits is fundamental with respect to selecting relevant therapeutic approaches, pharmacological treatments, or combinations of these. At present, few clinical measurement tools exist to diagnose attention deficits in children (e.g., Continuous Performance Test, Child Behaviour Checklist), and there are no methods available for diagnosis of primary attention deficit. The neuropsychological attention network test (ANT) may be useful in this capacity, but efforts are needed to investigate the performance of the test more carefully |
| Objectives | 7 | This systematic review seeks to address the research question: Do primary school-age children diagnosed with ADHD or at risk of ADHD demonstrate differences in the efficiency of functioning of the three attention networks measured with the ANT when compared to non-ADHD children? In answering this question, we will refer to differences in performance of children diagnosed with ADHD and at risk of ADHD in terms of alarm, conflict, and orientation networks compared to children without any ADHD symptoms. The following secondary questions were posited:  (1) Do primary school-age children diagnosed with ADHD or at risk of ADHD compared to non-ADHD children demonstrate differences in correctness scores (number of commission and omission) measured with the ANT?  (2) Do primary school-age children diagnosed with ADHD or at risk of ADHD compared to non-ADHD children demonstrate differences in the number of mistakes made in a particular type of clue or flankers measured with the ANT?  (3) Do primary school-age children diagnosed with ADHD or at risk of ADHD compared to non-ADHD children demonstrate differences in the ratio times in a particular type of clue or flankers measured with the ANT?  (4) Do primary school-age children diagnosed with ADHD or at risk of ADHD compared to non-ADHD children demonstrate differences in results of the ANT depending on the used version of ANT, e.g. LANT, ANT-R, ANT-C, ANT-I, ANTI-V? |
| METHODS | | |
| Eligibility criteria | 8 | **Participants**: We will include studies of individuals of primary school age, which according to the assumptions of The International Standard Classification of Education 2011 means the age between 5 and 13 years, both sexes without restriction to nationality, who had an ADHD diagnosis or were considered to be at high risk of ADHD. ADHD should have been diagnosed by a specialist (psychiatrist, clinical psychologist or any other qualification medical staff) based on DSM-5 (ADHD diagnostic code: 314) or based on either earlier versions of the DSM or the International Classification of Diseases (ICD, ADHD diagnostic code according to the ICD-11: 6A05). The high-risk group of ADHD includes children with symptoms of ADHD measured by ADHD symptoms questionnaires (like CONNERS-3, Structured Diagnostic Interview Questionnaire for ADHD - or foreign equivalents). Children with comorbidities (such as anxiety disorder, conduct disorder, learning disorder, and oppositional defiant disorder) will be included due to the nature of common coexistence with ADHD (8-10). However, other atypical concomitant or concurrent disorders (such as eating disorders, depressive or bipolar disorders, obsessive-compulsive disorders, or factitious disorders) will be excluded because of the potential contribution to the clinical symptoms and influence of AND performance.  **Intervention/Exposure**: The review will include studies regardless of the type of intervention for which the effectiveness was measured using the ANT.  **Comparator/Control**: We will include studies including individuals of primary school age, both sexes, without restriction to nationality, without ADHD symptoms or ADHD diagnosis.  **Outcomes**: Attention deficit measured by any version of the ANT, e.g. LANT, ANT-R , ANT-C, ANT-I, ANTI-V  **Study Type**: We will include prospective cohort studies addressing attention deficit measured with the ANT and prospective studies of intervention effects with a control group (both randomized and non-randomized controlled).  **Location**: We do not impose any restrictions on the area of the conducted research.The target population could be recruited from both primary schools or health care facilities (such as primary care settings, therapeutic settings, or diagnostic settings). |
| Information sources | 9 | The following electronic databases will be searched: PubMed, PsychInfo, Web of Science, EMBASE, DARE, and the Cochrane Library. Electronic database searching will be supplemented by hand-searching reference lists of the included review articles to identify any additional studies. The search will be not restricted to any language, sample size or year of publication. We will exclude editorials, letters, case studies, case series, and conference abstracts.. |
| Search strategy | 10 | Literature search strategies will be developed using Medical Subject Headings or equivalent and text word terms and words related to the nosological unit and the ANT. In addition, Boolean operators along with proximity operators (parentheses and quotations) for each database will be applied. The search strategy included terms relating to condition (attention deficit disorder with hyperactivity [MeSH] OR ADHD) and measurement tool (Attentional Network Test" OR "Attentional Networks Test" OR "Attentional Network Task" OR "Attentional Networks task" OR "Attention Network Test" OR "Attention Networks Test" OR "Attention Network Task" OR "Attention Networks task”). We have already piloted the initial search strategy (including searching terms and filters) for PubMed in March 2021 to investigate whether the searching strategy allows us to find potentially relevant reports. The pre-tested searching allowed us also to improve the search terms. |
| Study records: |  |  |
| Data management | 11a | One reviewer will search databases and handsearch the reference list of the included review articles. All potentially relevant records will be extracted to EndNote reference management software. At this stage, duplicates will be detected and deleted. After a year additional search will be done the same way. |
| Selection process | 11b | Two review authors will screen titles and abstracts for their eligibility for inclusion under the inclusion criteria providing the reason(s) for rejection. Any discrepancies at the screening stage will be resolved by discussion with another reviewer. The eligibility criteria will be pre-tested on a reasonable sample of reports. We will obtain full reports for all titles that appear to meet the inclusion criteria or where there is any uncertainty. Two review authors independently will then screen the full-text reports and decide whether these meet the inclusion criteria proving the reason for rejection. Assessment of the relevance of studies will be conducted by clinical psychology researchers expert in the content area. They will seek additional information from each study’s corresponding authors whenever it is necessary to resolve questions on eligibility. Eligibility criteria for each study will be assessed in order of importance, starting from participants, followed by the outcome, intervention/exposure, comparator/control and study design. Applying this strategy causes the first ‘no’ response to be the primary reason for excluding the study, and the remaining criteria will not be assessed. We will record the reasons for excluding studies. We will use a formal measure of an agreement to describe the extent to which assessments by two authors are the same. Disagreements at the assessment eligibility stage will be resolved by discussion with another reviewer. Multiple reports of the same study will be merged based on matching of the following study characteristics: author names, location and setting, numbers of participants and baseline data, and duration of the study. Where any uncertainties remain we will contact the corresponding authors. Neither of the review authors will be blind to the journal titles, or the study authors, or their institutions. The PRISMA template will produce a flow chart showing details of studies included and excluded at each stage of the study selection process |
| Data collection process | 11c | Two authors independently will extract data from the studies based on a specifically designed and pre-piloted data extraction form. Data extraction will be done by content area experts from the clinical psychology field who are familiar with the ANT, and who will be trained in how to code entries in the data collection form. Discrepancies will be resolved by discussion or/and consultation with another reviewer when needed. In case of discrepancies that cannot be resolved, we will contact the study authors; however, if this is unsuccessful, the discrepancies will be reported in the review. Corresponding authors will also be contacted to obtain any missing data. In case of multiple reports of the same study/project, we will extract data from each report separately and combine information across multiple data collection forms afterwards |
| Data items | 12 | The following information will be extracted from the studies:  • Publication details – title, author; year of publication; DOI number; country of a study conducting  • The number of participants per group  • Characteristics of the clinical population – age, sex, ADHD group type (ADHD/risk of ADHD), ADHD intensity evaluated by the results of the questionnaire (e.g., Conners 3); the sub-type of ADHD diagnosis (predominantly inattentive, predominantly hyperactive/ impulsive, and combined), diagnosis provider; diagnosis method(s); comorbidities, pharmacotherapy (yes/no); pharmacotherapy used during ANT assessment (yes/no)  • Characteristics of the control population – age, sex  • Study design - Prospective cohort study/intervention study  • The ANT results - Mean and standard deviation or median and range (or a standardized effect measures such as Cohen’s d) of the executive, alerting and orienting attention network, mean and standard deviation or median and range, as well as intra-individual variability of general reaction time. A number of omissions (missing answers) and a number of commissions (wrong answers) errors or if there will be a lack of that data, general correctness rate (percent of the correct answer).In observational studies with repeated measurement or intervention studies with several time points, we will always extract baseline data. The version of the ANT used, how the training of the ANT was performed, how the instructions were presented, the person conducting the test and their interventions with the child during the test, and any other descriptive data about the ANT performance and conducting.  • Characteristics of the interventions – types of intervention, frequency, duration. |
| Outcomes and prioritization | 13 | *Primary outcomes:* Mean and standard deviation or median and range (or a standardized effect measures such as Cohen’s d) of the executive, alerting, and orienting attention network, measured by the ANT.  *Secondary outcomes:* Mean and standard deviation or median and range and intra-individual variability (or a standardized effect measures such as Cohen’s d) of general reaction time achieved in the ANT. A number of omissions (missing answers) and a number of commissions (wrong answers) errors or if there will be a lack of that data, general correctness rate (percent of the correct answer) reached in ANT. |
| Risk of bias in individual studies | 14 | An assessment of potential bias will be done independently by two review authors. Any discrepancies will be resolved by arbitration among reviewers, together with content area experts from clinical trial methodology if needed. To assess the risk of bias in each included study, we will use the Revised Cochrane Collaboration Risk of Bias Tool (RoB 2) for randomized trials and the Risk Of Bias In Non-randomized Studies - of Interventions (ROBINS-I) tool for non-randomized studies. The risk of bias will be judged as high, low, or unclear risk bias. For cohort studies, we will use The Newcastle-Ottawa Scale, which assess the quality of cohort studies by a judgement of the selection of the study groups, the comparability of these groups, and the ascertainment of either the exposure or outcome of interest. |
| Data synthesis | 15a | If two or more studies are found to be sufficiently clinically and statistically homogeneous to be combined in a meta-analysis, we will pool the effect estimates of these studies using standard meta-analytical techniques described in the Cochrane Handbook for Systematic Reviews of Interventions. We will employ the fixed-effects estimator in the absence of materially important heterogeneity and the DerSimonian-Laird random effects estimator otherwise.  We will only combine studies for which the effect estimates can be converted to a common metric (e.g., group means and standard deviations into Hedges’s g).  We will not combine in the same meta-analysis studies of different design (observational and intervention studies). We will also not pool together multiple estimates coming from the same study or from statistical tests based on the same or overlapping subjects, as they cannot be considered independent, without taking within-study correlation into consideration. If a study reports effect estimates for the same outcome from more than one between-group tests, we will extract the estimate we believe provides more direct evidence or is less biased (e.g., is associated with a larger sample size or better adjustments) and will justify our decision to prioritize it. |
|  | 15b | Statistical heterogeneity in the models will be suggested by a significant Cochran’s Q at the p < 0.1 level and quantified by the I2 statistic as follows: mild (< 30%), moderate (30–50%) or high (> 50%). We will also inspect the direction of individual study effect sizes and the overlap of their confidence intervals.  Presence of publication bias for each outcome will be judged graphically and quantitatively. If the meta-analysis includes 10 or more studies, we will construct a funnel plot and test for asymmetry using Egger’s regression test [43]. Next, we will also generate a Doi plot, which plots study-level effect sizes against a rank-based measure of precision (z-score, where the midpoint is defined by the most precise studies and the less precise studies are scattered outward towards the tails of the plot) [44]. Asymmetry in a Doi plot can be tested with even 5-10 studies using an index called Luis Furuya-Kanamori (LFK) index, which is not a p-value based test, rather quantifies the difference between the two areas under the Doi plot curve created by the midpoint [45]. Major asymmetry will be indicated by an asymmetrical Doi plot and LFK index >\|2  Quantitative synthesis will be carried out using Stata v. 17 (College Station, TX: StataCorp LP.) and MetaXL v. 5.3 (EpiGear International Pty Ltd, Sunrise Beach, Queensland, Australia). |
|  | 15c | Sensitivity analyses:  Where applicable, we will conduct leave-one-out meta-analysis for each outcome to determine whether excluding studies one-at-a-time would materially change the pooled effect. This way we could identify influential studies  Given growing concerns about the appropriateness of the random effects model and its potential to yield overly liberal findings, we will also re-run the meta-analysis using the inverse-variance heterogeneity model; it was built under the fixed effect model assumption with a quasi-likelihood based variance structure to retain a correct coverage probability and yield more conservative pooled estimates regardless of heterogeneity  Subgroup analyses:  • ADHD diagnosis vs high-risk of ADHD vs non-ADHD child  • All results obtained with all versions of the ANT with one another  • Type A ADHD vs type B ADHD  • Symptoms of ADHD at intensity A against symptoms at intensity B |
|  | 15d | We will make a narrative synthesis of the findings from the included studies, structured around the participants (demographic and clinical characteristics), the ANT results, and characteristics of the interventions (in the case of intervention-based research), along with a comparative table. Quantitative data will be combined only if means and standard deviations are available or can be derived from available data. |
| Meta-bias(es) | 16 | NaN |
| Confidence in cumulative evidence | 17 | For each outcome, we will grade the quality of evidence using the Grading of Recommendations, Assessment, Development and Evaluations (GRADE) approach. Evidence will be judged as “high”, “moderate”, “low”, or “very low” quality depending on the extent to which we can be certain that the pooled effect estimate is close to the true effect. For randomized trials, we will start at “high”, and for observational studies, at “moderate” quality. The quality of evidence will be downgraded by 1 level for each of the following reasons – high risk of bias across the studies, indirectness of evidence (indirect population, intervention, control, outcomes), high heterogeneity (I2 > 50%) or inconsistency of results across studies, imprecision of results (wide confidence intervals, small sample size), and high probability of publication bias (presence of meaningful funnel plot and/or Doi plot asymmetry). Since there are no clear-cut recommendations on imprecision with continuous outcomes and standardized effect measures, we will downgrade if the sample size is < 620 (calculated under standard assumptions of α = 0.05, power = 0.80, and effect size of 0.20, and will consider the width of the confidence interval around the point estimate and the range of values it includes.  If there are no serious concerns about risk of bias, we may upgrade the quality of evidence by one level for large magnitude of effect, if a dose-response gradient is observed, and/or if accounting for all plausible confounding would reduce the pooled effect or suggest a spurious effect when results show no effect. We define a large effect according to Cohen’s convention of 0.80, although we recognize that this cutoff may be too strict or not equally relevant across psychological sub-disciplines. In addition, to upgrade for a large effect, the lower limit of the confidence interval of the point estimate will have to be at least 0.80 or greater. |

*** It is strongly recommended that this checklist be read in conjunction with the PRISMA-P Explanation and Elaboration (cite when available) for important clarification on the items. Amendments to a review protocol should be tracked and dated. The copyright for PRISMA-P (including checklist) is held by the PRISMA-P Group and is distributed under a Creative Commons Attribution Licence 4.0.**

*From: Shamseer L, Moher D, Clarke M, Ghersi D, Liberati A, Petticrew M, Shekelle P, Stewart L, PRISMA-P Group. Preferred reporting items for systematic review and meta-analysis protocols (PRISMA-P) 2015: elaboration and explanation. BMJ. 2015 Jan 2;349(jan02 1):g7647.*
