# Supplementary material for: Attention deficit in children with attention deficit hyperactivity disorder at primary school age measured with the attention network test (ANT): A protocol for a systematic review and meta-analysis
Source: PLoS One. 2022 Oct 25;17(10):e0275599. doi: 10.1371/journal.pone.0275599 (PMC9595530; doi:10.1371/journal.pone.0275599)
Supplement: Attachment — Submitted filename: Response to Reviewers-2.docx [file pone.0275599.s003.docx]

**PONE-D-22-06141**

**Response to reviewer comments**

**TITLE:** Attention deficit in children with attention deficit hyperactivity disorder at primary school age measured with the attention network test (ANT): a protocol for a systematic review and meta-analysis

**REVIEWER 1**

Reviewer #1: This is a very interesting study and a relevant topic for the scientific community. In addition, the authors have made all the requested changes

***RESPONSE:* Thank you for the evaluation of our revision. Your kind words are deeply appreciated.**

**REVIEWER 2**

Reviewer #2: The authors have carefully addressed all suggestions. They even have added information (e.g. data extraction) that properly complemented the protocol.

Just one minor issue regarding my question about meeting abstracts. Some databases retrieve abstracts from conferences or international meetings, which sometimes provide data. I consider it is important to state if only abstracts from published, peer review papers will be selected or also abstracts from conferences.

***RESPONSE:* We would like to thank you for your time to review our revised manuscript and for your kind words. Although we had considered it, due to the possibility of gathering more data, we decided not to include the conferences' abstracts. The conferences or international meetings' abstracts, even if containing information about the results, are not providing enough information to verify their study. Furthermore, that was repeatedly proven, that data from the conference abstracts cannot be trusted and the quality of their studies is hard to estimate^[1]^.**

**For clarification, in our manuscript, under the Search Strategy section we edited the following sentence:**

**“The search will be not restricted to any language, sample size, or year of publication. We will exclude editorials, letters, case studies, case series, and conference abstracts.”**

1. E.g. Yoon, U., Knobloch, K. Assessment of reporting quality of conference abstracts in sports injury prevention according to CONSORT and STROBE criteria and their subsequent publication rate as full papers. BMC Med Res Methodol 12, 47 (2012). https://doi.org/10.1186/1471-2288-12-47
